# Bicuspid Aortic Valve Endocarditis and Q Fever

**DOI:** 10.1016/j.jaccas.2025.103771

**Published:** 2025-06-18

**Authors:** Sara J. King, Katherine V. Trinh, Masataka Kawana

**Affiliations:** a Department of Medicine, Stanford University School of Medicine, Stanford, California, USA; b Division of Cardiovascular Medicine, Stanford University School of Medicine, Stanford, California, USA

**Keywords:** bicuspid, culture-negative, endocarditis, Q fever

## Abstract

**Background:**

Patients with bicuspid aortic valves (BAVs) are at increased risk of infective endocarditis. *Coxiella burnetii* is a rare cause of culture-negative infective endocarditis.

**Case Summary:**

A young male with BAV presented with 3 months of fevers and abdominal distension. Initial workup showed pancytopenia and massive splenomegaly. Blood cultures were negative. Echocardiography revealed endocarditis of the bicuspid valve, aortic regurgitation, and aortic pseudoaneurysm. He underwent annulus debridement, valve replacement, and concurrent splenectomy. Serologic and tissue studies revealed *C burnetii* and Q fever.

**Discussion:**

Chronic Q fever is a clinical syndrome that presents months to years after *C burnetii* infection with nonspecific symptoms. Due to its intracellular and fastidious nature, treatment requires a prolonged antibiotic course and standard surgical indications for infective endocarditis.

**Take-Home Message:**

Patients with BAVs are at increased risk of endocarditis, and *C burnetii* should be considered in all cases of culture-negative endocarditis.

## Visual Summary

**Figure:**
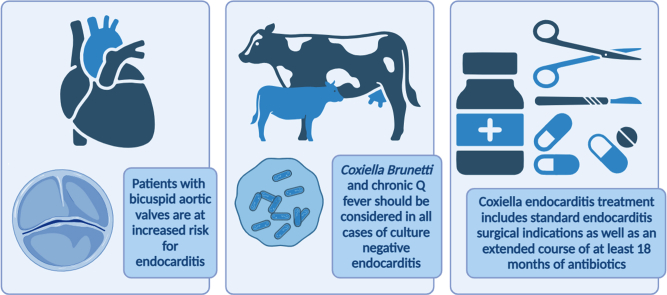

## History of Presentation

A young male in his 20s with a known bicuspid aortic valve (BAV) presented to an outside hospital with 3 months of nightly fevers and worsening abdominal distension. He was found to have pancytopenia and splenomegaly concerning for hematologic malignancy and echocardiogram findings concerning for endocarditis of the BAV. The patient was transferred to our center for a higher level of care. On presentation he was afebrile, with a heart rate of 158 beats/min, blood pressure of 122/90 mm Hg, and SpO2 of 100% on room air. Physical examination was notable for regular tachycardia, sharp S2, and visible carotid pulsations with sharp upstroke and rapid downstroke. The abdomen was distended with palpable tender splenomegaly. He had trace lower extremity edema. The patient reported the removal of 4 impacted wisdom teeth 3 months before his presentation. He did not take antibiotic prophylaxis before his dental procedure per his cardiologist’s recommendations. He lives next to his parent’s farm with livestock.

**Take-Home Messages**

• Patients with BAVs are at increased risk of IE from both atypical and common odontogenic organisms, although these patients do not qualify for IE antibiotic prophylaxis per the 2007 AHA guidelines.
• One must maintain a high suspicion for *Coxiella* endocarditis and chronic Q fever in patients with valvular abnormalities and animal exposure because they may present with nonspecific symptoms with mimickers and negative blood cultures.

## Past Medical History

BAV with last echocardiogram 9 years before presentation that showed mild aortic stenosis, mild left ventricular hypertrophy, and moderate aortic regurgitation. He does not take any medications.

## Investigations

**Figure 1:**
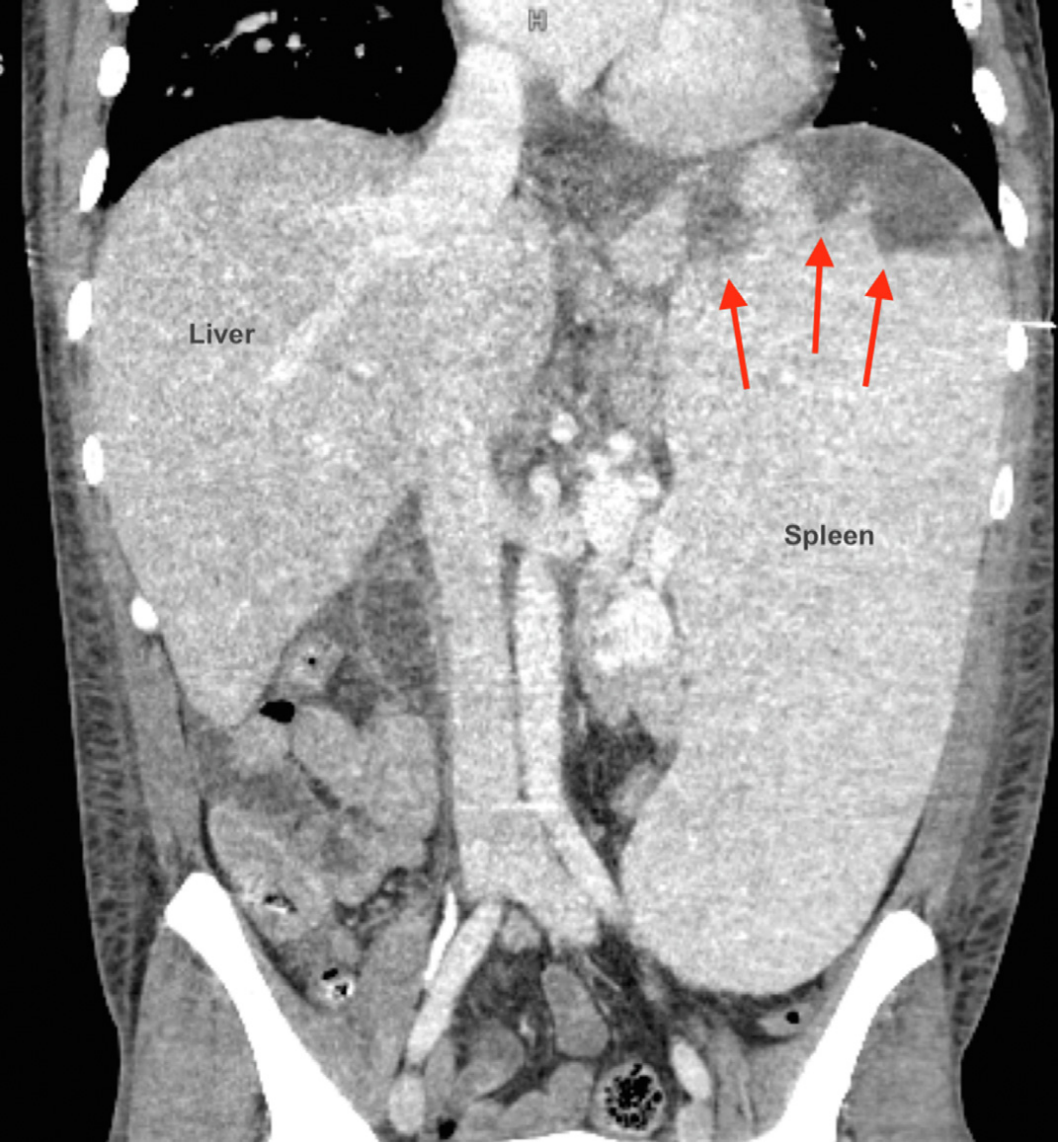
Computed Tomography Angiography of the Abdomen and Pelvis Computed tomography angiography of the abdomen and pelvis on admission showing massive splenomegaly, large wedge-shaped infarcts of the anterior and posterior pole (red arrows), and dilated portal vein suggesting portal hypertension (not pictured).

**Figure 2:**
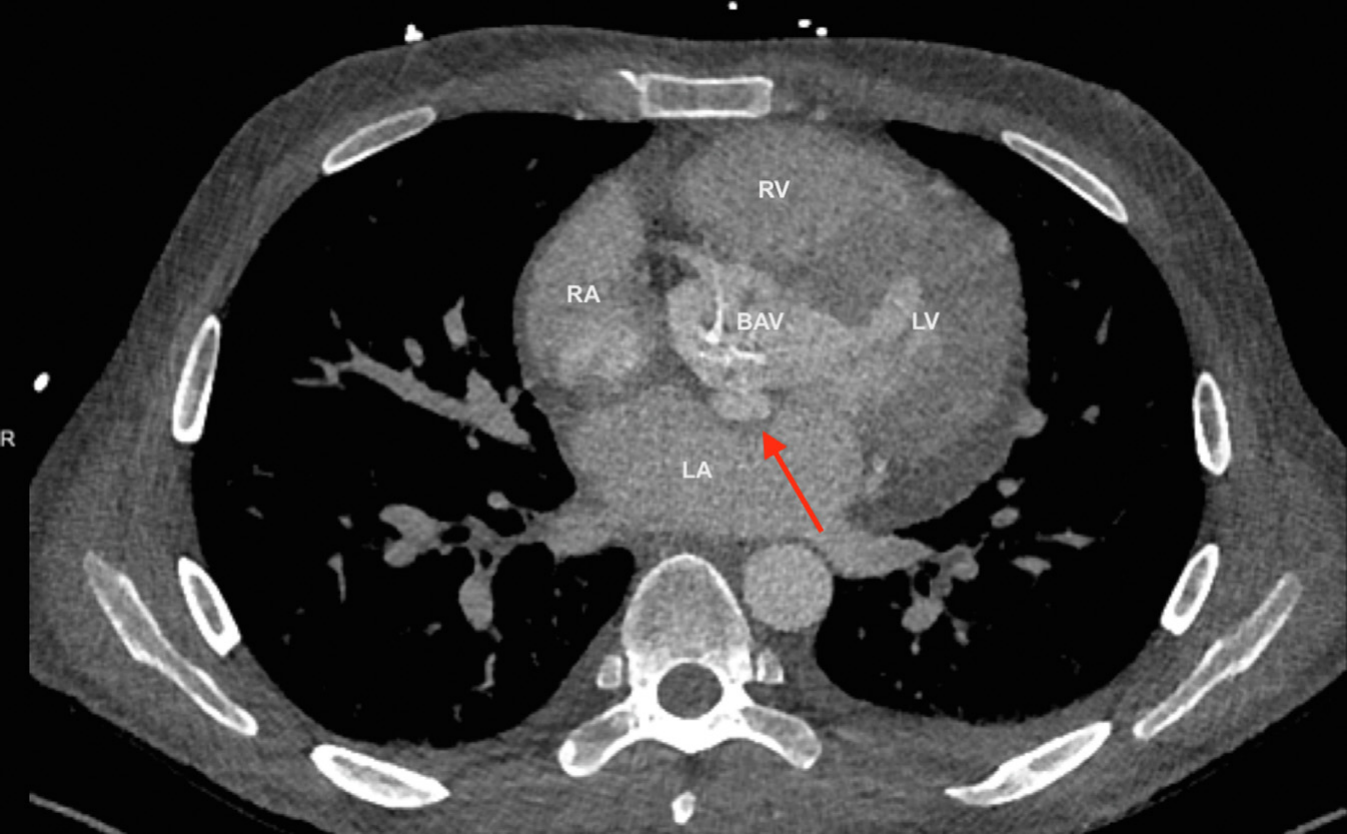
Computed Tomography Angiography of the Chest CT angiography of the chest showing an aortic root pseudoaneurysm (red arrow) arising just inferior to the junction of the noncoronary cusp and left coronary cusp projecting toward the aortomitral curtain. BAV = bicuspid aortic valve; LA = left atrium; LV = left ventricle; RA = right atrium; RV = right ventricle; other abbreviations as in Figure 1.

Laboratory tests revealed pancytopenia with a white blood cell count of 1.3 k/μL, differential with absolute neutrophil count of 0.68 k/μL, absolute lymphocyte count of 0.55 k/μL, hemoglobin of 9.1 g/dL, and platelets of 86 k/μL. Comprehensive metabolic panel with creatinine of 1.17 (estimated glomerular filtration rate: 87; unknown baseline), aspartate aminotransferase of 168, and alanine aminotransferase of 72. The patient had mildly elevated thyroid stimulating hormone at 4.41 mIU/L with normal T4. The patient also had a N-terminal pro–brain natriuretic peptide of 24,598 pg/mL and high-sensitivity troponin of 24 ng/L with 4-hour trend to 25 ng/L. Electrocardiogram displayed sinus tachycardia at a rate of 155 beats/min. Chest x-ray showed trace pleural effusions, with borderline cardiomegaly. Bedside echocardiography revealed a large mass on the BAV with aortic valve prolapse and severe aortic regurgitation. Computed tomography of the chest, abdomen, and pelvis displayed an aortic root pseudoaneurysm, massive splenomegaly (32 cm) with wedge-shaped infarcts of the anterior and posterior pole, and a dilated portal vein suggesting portal hypertension (Figures 1 and 2).

## Management

**Figure 3:**
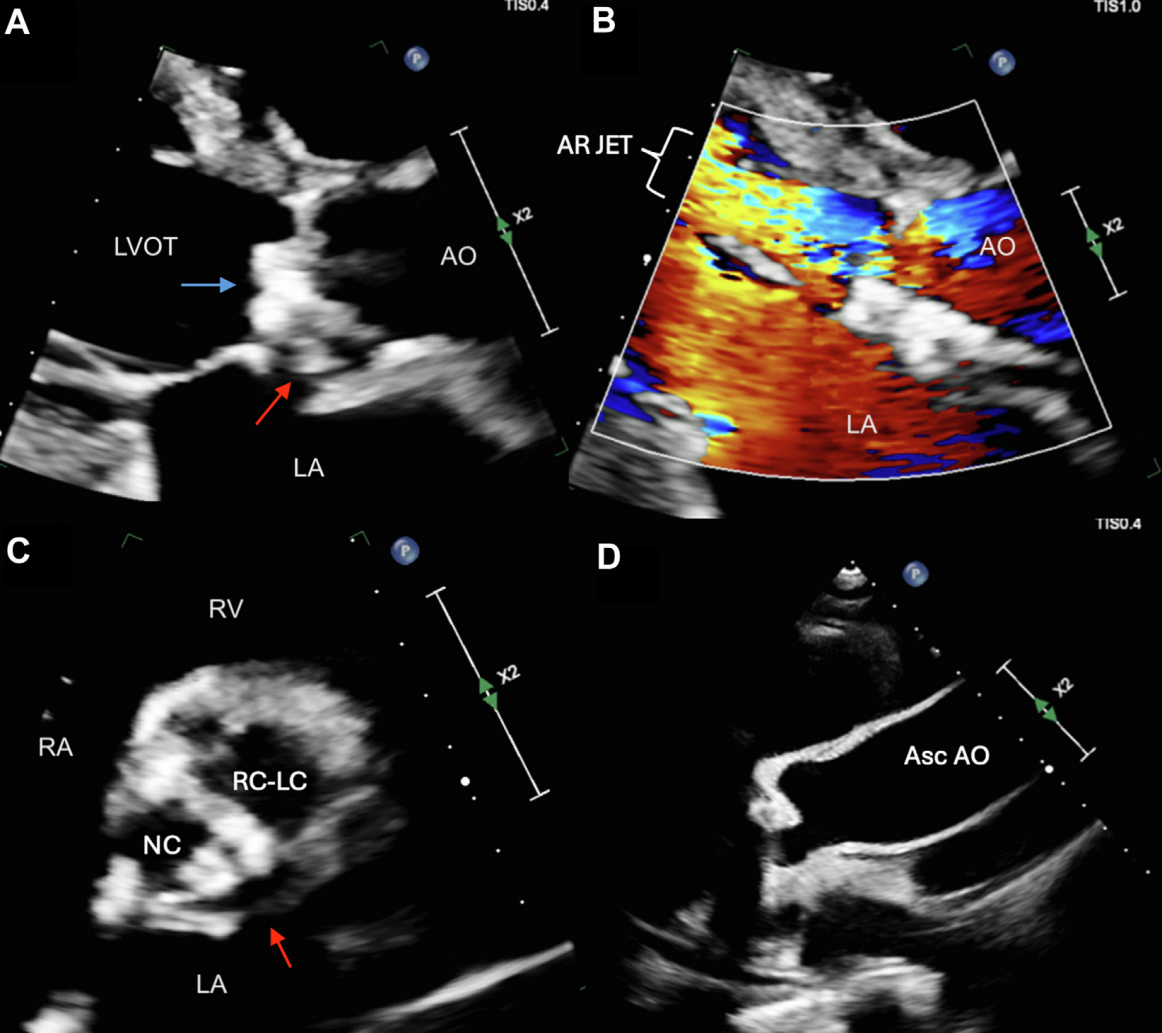
Transthoracic Echocardiogram Transthoracic echocardiogram showing: (A) parasternal long zoomed in view of the BAV with vegetation (blue arrow) and pseudoaneurysm (red arrow). (B) Parasternal long zoomed in view of the BAV with doppler showing aortic insufficiency regurgitant jet (white bracket). (C) Parasternal short zoomed in view of BAV with fused RC-LC and NC with vegetation and pseudoaneurysm (red arrow). (D) Parasternal long view of dilated proximal Asc AO (4.4 cm). AO = aorta; Asc AO = ascending aorta; AR JET = aortic regurgitation jet; LVOT = left ventricular outflow tract; NC = noncoronary cusp; RC-LC = right and left coronary cusp; other abbreviations as in Figure 2.

**Figure 4:**
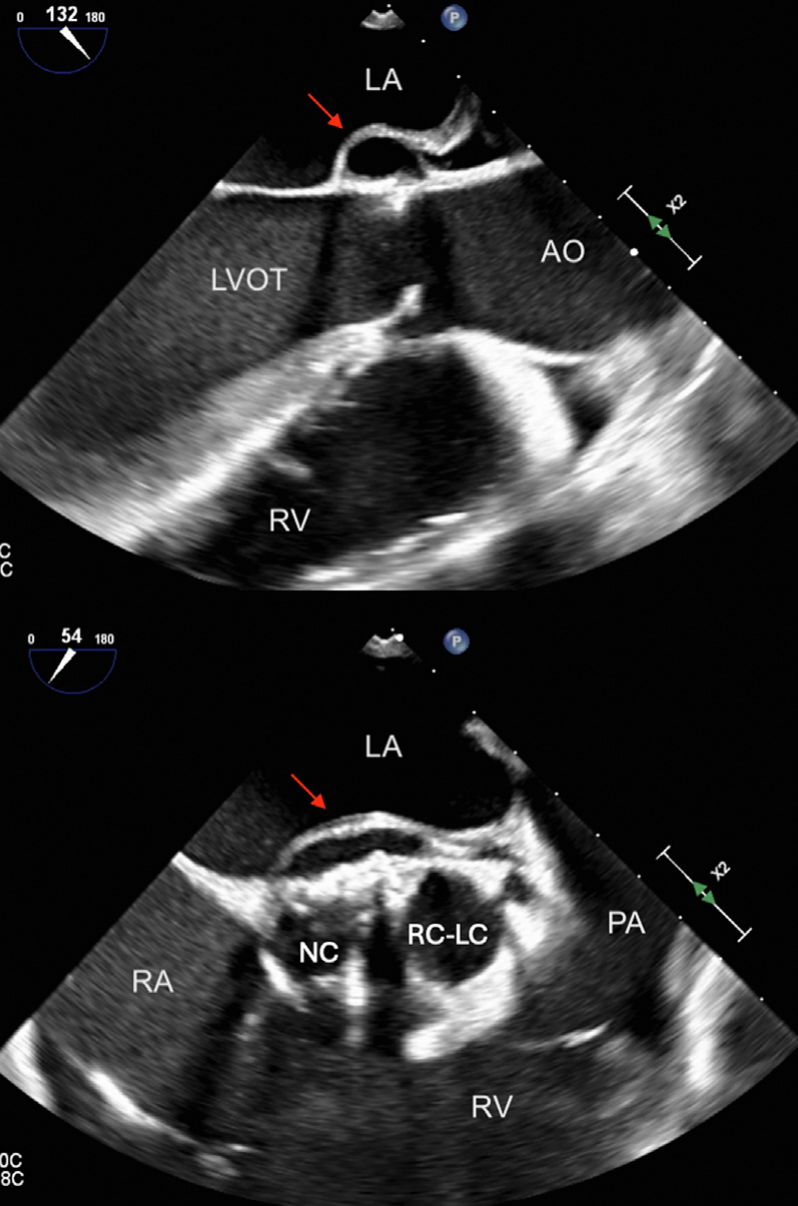
Intraoperative Transesophageal Echocardiogram Intraoperative transesophageal echocardiogram showing BAV with RC-LC fusion with vegetation, NC, and pseudoaneurysm (red arrows). PA = pulmonary artery; other abbreviations as in Figures 2 and 3.

**Figure 5:**
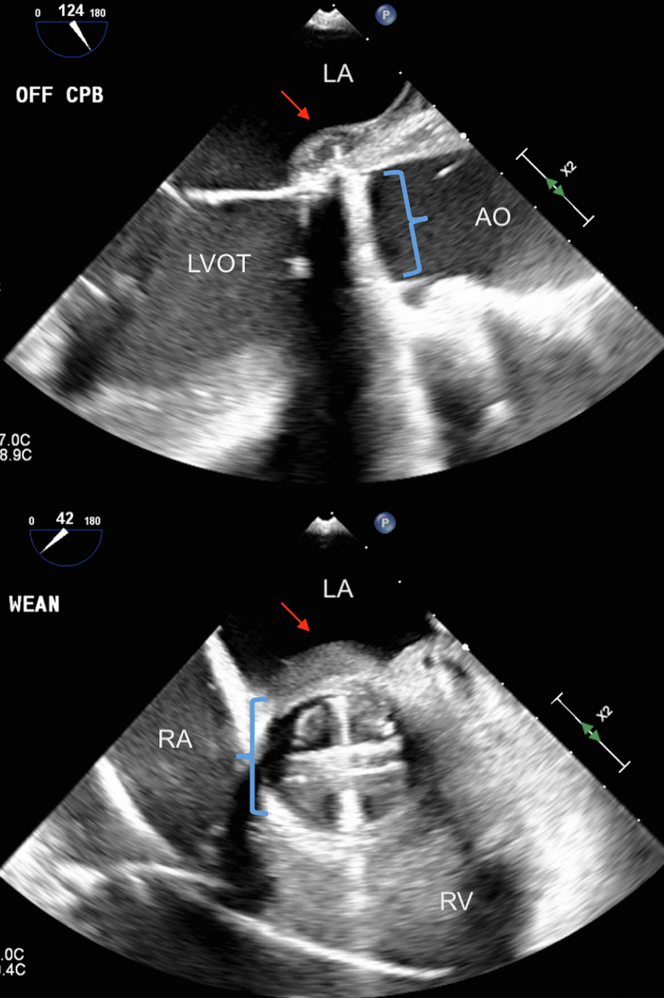
Intraoperative Transesophageal Echocardiogram Intraoperative transesophageal echocardiogram after 25-mm On-X mechanical aortic valve replacement (blue brackets) and pseudoaneurysm patch (red arrows). Abbreviations as in Figures 2 and 3.

He was admitted to the cardiac intensive care unit for close monitoring given the degree of aortic regurgitation and risk of decompensation. Blood cultures were obtained, and the patient was initiated on intravenous vancomycin and cefepime. A formal echocardiogram was performed, which displayed the mentioned findings, as well as moderately reduced left ventricular ejection fraction at 37%, moderate left atrial enlargement, mild right atrial enlargement, mild right ventricular enlargement with mildly reduced function (right ventricular fractional area change 24%), and a dilated and noncollapsible inferior vena cava (Figure 3). Cardiothoracic Surgery was consulted, and, given the concerns for pseudoaneurysm with severe aortic valve damage with prolapse and newly reduced ejection fraction, planned for urgent surgery. However, the patient’s pancytopenia and massive splenomegaly raised concerns regarding postoperative bleeding. General Surgery was consulted and agreed to perform a concurrent splenectomy. The patient was taken to the operating room the following day, where he first underwent laparotomy and splenectomy, followed by cardiac surgery. Intraoperatively, he was found to have a destroyed noncoronary cusp with a pseudoaneurysm opening just above the aortomitral curtain with a significant sac, however, without evidence of active infection or pus (Figure 4). Given these findings, the pseudoaneurysm sac was excluded with annular stitches, and there was no need for aortic root replacement. The annulus was debrided, and the patient had a 25-mm On-X mechanical valve placed (Figure 5). Splenic tissue was sent for pathology and flow cytometry. Aortic valve tissue was sent for pathology, cultures, and bacterial sequencing.

During the patient’s admission, Infectious Disease was consulted for infective endocarditis (IE). Blood cultures and aortic valve tissue cultures had no growth throughout admission. Culture-negative endocarditis workup was sent, including serum *Coxiella*, *Bartonella*, and *Brucella* antibodies, which resulted with Coxiella antibody titers of immunoglobulin M 1:64 and immunoglobulin G >1:32,768. Bacterial identification using DNA sequencing confirmed *Coxiella burnetii* from the aortic valve tissue. The patient was transitioned from vancomycin and ceftriaxone to doxycycline and hydroxychloroquine for a planned 18- to 24-month course with outpatient Infectious Disease follow-up.

In addition, Hematology had been consulted for his pancytopenia with massive splenomegaly due to concern for underlying malignancy. Blood smear was negative for blasts or malignant cells, and flow cytometry on both serum and splenic tissue showed no evidence of underlying hematologic malignancy. JAK2 and FISH for BCR-ABL were negative, and a broad nutritional and further infectious workup was also unrevealing. Spleen pathology showed expansion of the red pulp, dilation of the veins and sinuses, and large areas of infarction with relative preservation of the red pulp, altogether consistent with congestion from elevated portal pressures from the patient’s aortic regurgitation with splenic sequestration and septic emboli.

## Outcome and Follow-up

The patient had an uncomplicated recovery from his splenectomy and mechanical aortic valve replacement. His pancytopenia resolved over the ensuing days, consistent with pancytopenia due to splenic sequestration. He was initiated on warfarin for his mechanical valve. He was discharged 10 days after his initial presentation with Cardiothoracic Surgery and Infectious Disease follow-up. At present, he continues to take doxycycline and hydroxychloroquine for his Q fever IE.

## Discussion

BAV is the most common congenital heart disease, estimated to impact between 0.5% and 2% of the U.S. population.^1^ The pathophysiology for increased IE risk is thought to be increased shear stress across the BAV, which over time may lead to endocardial tissue damage and increased susceptibility to infection.^2^ The most commonly implicated organisms are odontalgic in origin, including the *Streptococcus viridans* group, other *Streptococcus,* and *Staphylococcus* organisms.^3^ However, up to 11% of IE cases are culture-negative, which may be due to antibiotic administration timing, fastidious organisms, or intracellular organisms that are not readily cultured, such as *Bartonella, Chlamydia,* and *Coxiella* species.^3^^,^^4^ Here, we present a rare case of *C burnetii* endocarditis and chronic Q fever in a patient with a BAV.

Chronic Q fever is a clinical syndrome that presents months to years after *C burnetii* infection, most often with endocarditis, and is associated with high mortality if left untreated.^5^ It is estimated to impact 1%-5% of those infected with acute Q fever, and, while exceedingly rare, the incidence of chronic Q fever has increased in the last decade in parallel with acute Q fever.^5^^,^^6^ This may be related to increasing environmental hazards and overcrowding because it is acquired from inhaling the aerosols of infected animal parturient, most often from cattle, sheep, and goats.^6^ Chronic Q fever presents with nonspecific symptoms of fever, malaise, anorexia, progressive weight loss, cytopenias, and, rarely, hepatosplenomegaly.^5^ Given these nonspecific symptoms, chronic Q fever is often missed or mistaken for other diagnoses; in our patient’s case, he was initially told at the outside hospital that he likely had a hematologic malignancy. As previously mentioned, *C burnetii* is not readily cultured in blood given its intracellular nature. Instead, polymerase chain reaction and serologies of serum and/or infected tissue, along with imaging (echocardiography or positron emission tomography–computed tomography), and a consistent clinical presentation can confirm the diagnosis.^5^ In fact, the Duke criteria was revised in 2000 to include significantly elevated *Coxiella* serologies as a major criteria.^6^ Treatment is prolonged given the organism’s fastidious nature, and consists of a minimum of 18 months of doxycycline and hydroxychloroquine with monthly monitoring of serologic titers for response.^6^ Surgical indications are the same for Q fever endocarditis as other cases of IE etiologies, including severe valvular dysfunction causing heart failure and complications such as abscess or pseudoaneurysm, among others.^7^

Although blood tests and pathology clinched the diagnosis of Q fever in our patient, there was initial concern for an odontogenic source of infection given the chronology of his teeth extractions and symptoms. The patient had discussed antibiotic prophylaxis with his cardiologist before his procedure, and, in concordance with current American Heart Association (AHA) guidelines, he was not recommended antibiotic prophylaxis. The use of antibiotic prophylaxis for IE is a topic of debate. In 2007, the AHA modified the indications for IE antibiotic prophylaxis to exclude intermediate-risk patients, including patients with BAV, due to lack of evidence for antibiotic prophylaxis preventing IE cases and the risk of antibiotic-associated adverse events, including greater drug resistance.^8^ Studies have demonstrated no change in IE trends overall in the United States and Canada after the 2007 AHA guideline change.^9^^,^^10^ However, there is little data specifically on antibiotic prophylaxis in BAV patients, and 1 recent study demonstrated increased odontogenic IE risk in BAV patients, arguing for reconsideration of antibiotic prophylaxis in this population.^3^ Further research is needed to assess the risks and benefits of prophylactic antibiotics in patients with BAV.

## Conclusions

Our case demonstrates the presentation of a severe case of culture-negative endocarditis in a patient with a BAV. It exposes *C burnetii* as a rare, but increasing cause of IE, and describes chronic Q fever as a clinical syndrome with nonspecific syndrome requiring prolonged antibiotics. Finally, it highlights the increased risk of IE in patients with BAV, and emphasizes the importance of multidisciplinary care, including the involvement of cardiothoracic surgical teams and infectious disease specialists in the diagnosis and management of IE.

**Visual Summary:**
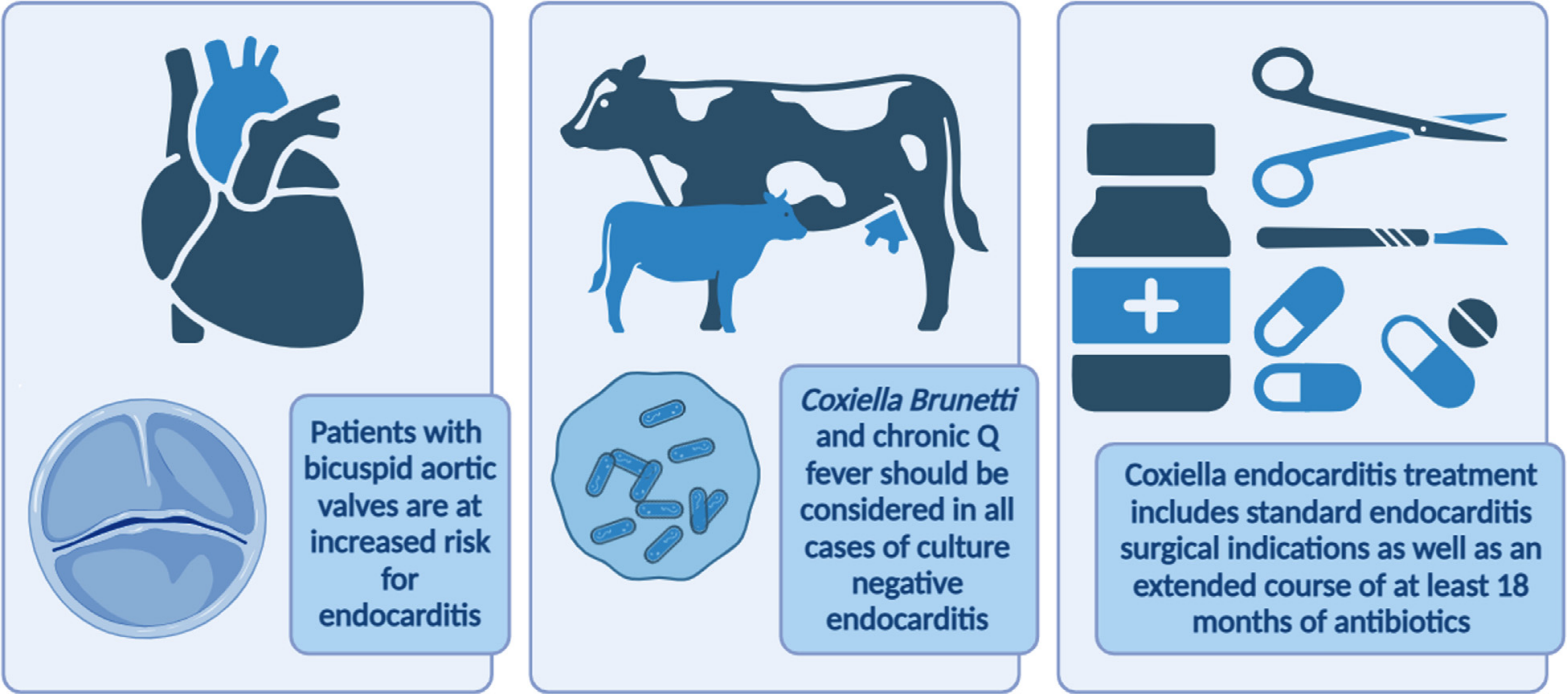
Bicuspid Valve Endocarditis and Q Fever Created using BioRender: Trinh K, 2025, https://BioRender.com/k83n711.

## Funding Support and Author Disclosures

The authors have reported that they have no relationships relevant to the contents of this paper to disclose.
